# Elements of Success in Dental Practice

**Published:** 1889-07

**Authors:** S. B. Palmer

**Affiliations:** Syracuse, New York


### International Dental Journal.

Vol. X. July, 1889. No. 7.

### Original Communications.[1]

### ELEMENTS OF SUCCESS IN DENTAL PRACTICE.[2]

BY S. B. PALMER, M.D.S., SYRACUSE, NEW YORK.

As we look out upon nature and see the globe with its mountains and valleys, its oceans and rivers and all the forms of life therein contained, together with the atmosphere that surrounds it, we can hardly comprehend that in nature's laboratory are found less than seventy simple elements from which all natural substances are made.

This illustration applies well to the practice of dentistry. A few positive elements harmoniously combined will insure success, as a few negative elements mechanically compounded will beget failures.

Elements combine according to the law of affinity in systematic order, and in definite proportions: thus in office practice we may see the end from the beginning, or anticipate results without experiments. We cannot change the relations between cause and effect, but we may so arrange causes that the right effects will follow. This is science: without this knowledge we must do as we are told, and good results are simply good luck.

Success in dental practice is made up of elements arranged by the

---

[1] The editor and publishers are not responsible for the views of authors of papers published in this department, nor for any claim to novelty, or otherwise, that may be made by them. No papers will be received for this department that have appeared in any other journal published in this country. The journal is issued promptly on the 15th of the month.

[2] Read at the semi-annual meeting of the Massachusetts Dental Society, Boston, June 5, 1889.

practitioner. Patients are retained or repelled according to the affinity between the operator and his patient.

Dentistry is a poor profession to choose without natural adaptation. If the inborn mechanical element is lacking, both dentist and patient will suffer in consequence. That dentist belittles himself who in after-years quotes his college instructions as authority for practice. The progressive dentist very soon outgrows his school-day teachings, and goes on thinking, acting, and inventing for himself.

Dentists may be divided into three classes; individuals are not necessarily confined to either from public sentiment; the boundaries are not arbitrary, but each finds his level according to the law of professional gravity.

It is often remarked that there is plenty of room at the top; so is there plenty of low standing-room at the bottom, which is well filled up with a professional mass rather than a compound. There may be found the coarser elements of the profession, such as have no affinity for higher grades, and who are seldom seen to rise above their level. This class receives support from a corresponding grade of patients who do not appreciate good work.

The second grade is mainly composed of those whose ambition is satisfied with a large practice and small profits. The management seems more mechanical than professional, although many destined for the highest standing are compelled to enter and pass through this training grade. For instance, the young graduate, without means to establish a lucrative practice, perhaps, too, in debt for his tuition, finds this the only starting-point, but with a proper combination of elements he will soon rise above and enter professional service. Here, too, may be found the established practitioner who does not dare to close his office in order to attend conventions, for fear that his competitor will secure the ten-dollar case he might lose by his absence. Others fail from lack of enthusiasm. Emerson says, "Without enthusiasm no great thing is accomplished." Thus they are held back by indifference.

The third degree is progressive in its nature, and is the only true representative caste of the profession. It advances practice, holds conventions, maintains literature, and establishes colleges. A combination of certain elements will lead to progress. Purity, frankness, and integrity lie at the base of moral character. Sacred as these virtues are, common sense is necessary in their application. It is desirable and important that every dentist gain the confidence of his patients. The young man will, of course, meet with cases beyond his skill to diagnose; frankness might prompt him to say, "I don't know." This, however true, would be an element of weakness; good judgment would meet the case by doing something promptly, and thus gain time to learn what to do.

On the other hand, the violation of the principles of integrity is sure to work against success. In the treatment of children no one can afford to inflict pain through deception. The breaking of a root in extracting may remain concealed from the patient's knowledge for months, but, when the fact is revealed, the sufferer is not usually in a forgiving mood, and confidence is at once destroyed.

Operations are often called for quite too difficult to be undertaken by the inexperienced. Never say the thing cannot be done; it is only a challenge for another to try, and, if the trial proves a success, the one is elevated, the other depressed in the estimation of the patient.

The fact that dentistry is being divided into specialties affords an inexperienced operator the opportunity to refer any operation he feels himself incapable of doing to a specialist. By so doing, the one referred to is flattered into friendship, while, on the other hand, the case might be turned to disadvantage.

Dignity is an important factor in character: rightly used, like iridium, it stiffens the compound, but too much renders it fragile. Reputation should be established upon real merit: to build upon the imperfections and failures of others is to place enemies at the foundation of your own success. Much harm comes of unnecessarily speaking ill even of disreputable dentists; they exert influence for good or evil.

In relation to education, it is well to invoice knowledge and know where we stand. Tabulate all new departures and the results of experiments. Knowledge is obtained from many sources; without some systematic method of retaining what is learned, one forgets a portion, and thus progress is slow. Practically, the reading of two or three journals will keep a student well informed. This benefit is due to patients.

Of course, at the present time no one should engage in dental practice without a college diploma. This, at once, is evidence of a certain amount of study; it introduces the holder to the public as well as to the profession. During college training, the mind is disciplined, manners are polished, instruction received in conducting the affairs of the office and conduct towards patients that private study never affords.

This is an essential element, but the dentist who relies wholly upon his college attainments and experience in his own practice is like a plant growing in a pot; the best possibilities are circumscribed. What lectures are to the student during his college course, discussions and clinics are to the dentist in practice. Reading is well, but the same communication imparted by enthusiastic speakers and the discussions which follow make impressions far more lasting. Seeing is believing; the instruction imparted in clinics cannot be successfully reported. Some think they cannot afford these opportunities. Gentlemen, it is stock in trade; we get nothing good without exertion; perpetual motion is still a dream of the future, because all motion requires energy. The formation of a crystal, the opening of a flower, the highest attainments of human intellect, are the results of forces spent and lives extinct. The dentist who does not draw from this source of knowledge will have none to spare for the benefit of his practice.

Dentists in every State or Province should be united in one brotherhood; condemnation and ill-treatment of others generally comes from ignorance,—not wholly from the want of knowledge respecting the usage of good society, but ignorance of the good qualities of the accused, which are best learned by friendly association. Again, it is unsafe to operate outside of professional protection. In this particular, union is strength.

In every community there are to be found would-be victims of malpractice. Equally shameful is the fact that the legal profession furnishes coworkers, ready to share in any black-mail speculation that can be made to pay.

All cannot possess an extensive library, but by care much may be done by way of a substitute. Some writer has remarked that if he could have but three books, they would be the Bible, Scientific American, and Index Rerum. I would make it four, and add an Unabridged Dictionary. A library index in the hands of the student would represent a library relating to the subjects most prominent in the mind of the compiler, as the book is an index of all knowledge entered therein or tells where the item or passage may be found, perhaps in an accompanying scrap-book or in books belonging to other libraries; all is there filed for ready reference.

We now leave the question of education and take up the subject of practical office work, from a scientific stand-point,—not that I have any new mode of operating to offer; practice and observation will instruct any one. Progress by this method is slow and empirical. Advancement in dentistry to the present is mainly the outcome of this method of teaching. Science that only demonstrates facts previously obtained is of no great benefit, as facts would stand without science. This movement reminds one of the crawfish, which travels backward as well as forward. The real benefit of science is to look ahead and be able to connect cause and effect. The result of operation in filling teeth is based upon positive natural laws or principles. Every filling when inserted becomes a cause; the effect may be concealed in the long future.

We desire to effect preservation by filling, and often fail. When we become conversant with the relations of the filling materials to the teeth filled, we may with certainty anticipate results, and thus introduce those conditions which will favor success. As an illustration, most operators have observed that gold fillings fail when inserted in children's teeth, especially in proximate cavities in the incisors. Practice has taught this, as well as that gutta-percha preserves this class of teeth until they are in a normal condition. One of the greatest mistakes of young operators is to fill this class of teeth with gold, thinking other failures were due to imperfect manipulation, when in fact there is a principle against success under these conditions, and repeated attempts are useless. The reasons will be given further on.

This doctrine was given to the profession some seventeen years ago, and for a number of years was discussed and not infrequently ridiculed, because it preferred charges against gold, which at that time was king, and to speak of facts was treason. Now gold and tin, gold and amalgam, and even copper amalgam, receive free discussion by leading dentists throughout the country.

Claiming nothing for contributions upon this subject, I will say that practice is greatly modified; the doctrine is incorporated in text-books and taught in college. The whole subject may be found in the second volume of the "American System of Dentistry."

A thorough knowledge of the adaptation of filling materials to the various conditions and kinds of teeth is an important element to success; we will therefore consider some of the conditions met with, and the means used in their treatment. Eighty per cent. of teeth that require filling may be disposed of in a few sentences, as all are familiar with the material used, as, all things considered, gold is best in these cases. We have no need to cite its success for forty or fifty years as proof that gold preserves teeth. On the other extreme, we can as truly say it will fail in two years; both are true. What we most need in dental education is clear and distinct ideas of principles. There are as many causes as there are definite results. For the sake of clearness, we will say that normal tooth-structure is composed of seventy-two parts lime or inorganic matter, which is almost a non-conductor of thermal or galvanic disturbances. Empiricism has long since established the fact that gold will preserve teeth of average density as long as needed. Let the argument be based upon this foundation, that all teeth may be filled with gold if the structure is sufficiently dense to exclude fluid circulation between the filling and the dentine, which is a well-established fact.

How are we to know this condition and what happens if there is a too large percentage of organic matter? The excavator reveals the character of the dentine as effectually as a probe does the nature of a wound. Dentine that resembles cartilage in structure, to a degree that cuttings are removed in shavings and without the noise given in cutting bone, cannot retain gold fillings long enough to do the dentist credit. This is so in particular of children's teeth, where lateral cavities in the incisors are evidences of immature development. When such teeth are filled, the gold is in contact with living tissue. In the fully-developed tooth the fillings rest mainly upon the mineral portion of the tooth.

In dentine, as in the soft tissues, excessive inflammation destroys vitality of the adjacent parts. Slight excitement stimulates, and nature repairs the injury by calcific deposits. Thus, teeth may be sensitive to thermal changes for weeks and months after filling and finally become comfortable, and the filling will be perfect; but, if there is not sufficient inorganic matter to protect the tissue from any changes injurious to it, inflammation destroys vitality instead of stimulating the pulp to make repairs.

In this case the devitalized surface turns dark and shows through the enamel back of the gold. In a short time the filling must be removed, and, if the dentine is found in no better condition, similar results may be anticipated from another gold filling.

I wish to be understood upon this point: there is a law of success under the first-named condition and a law of failure under the second. This was the fault of discussions upon the so-called "Electro-Chemical Theory." The opponents always chose the conditions to favor their case, and, being true, they had weight with all who could not see the perversion. It may be of interest to state that all teeth of low grade or highly organized ought to be filled with gutta-percha or some non-conducting material until sensitiveness is removed by deposition of lime salts. This may require months, and, in the case of children, years.

I wish to be emphatic upon another point respecting the failure of gold, which is due to defective manipulation and not to the material or dentine; that is, a leaky gold filling will injure good, sound dentine. Any one can recall the sensitiveness of dentine when poor or leaky gold fillings are removed. Without going into the scientific reasons for this fact, we will say that fluids which surround leaky gold fillings are acid and continue so, which of course dissolves the lime, thus leaving the organic matter exposed and subject to inflammation and subsequent decomposition. It requires but one more process to place a normal tooth upon the same plane as an abnormal one: when the mineral element is taken out, the dentine is in the same condition as a tooth that never possessed that property. This is the secret of success with some in the use of gold: some do perfect work; others cannot. Again, excessive malleting of cohesive gold upon frail dentine will bruise it, and thus give the opportunity for fluid action and secondary decay. For this reason, non-cohesive gold gives the best result as a lining in the hands of most operators. Let it be understood that gold possesses no antiseptic properties like tin and copper amalgam; in its use, perfect adaptation is the only salvation.

I will not add to the length of this paper by considering all the materials used for filling. Once the principle is understood, it may be applied to all the materials. The way out of these difficulties is to come through lining cavities with some material like varnish, in a manner to become a substitute for the lime dissolved.

In using amalgam, if the cavity is near the pulp, or very sensitive, I use gutta-percha stopping dissolved in chloroform. Having the amalgam ready, paint the cavity with the chloro-percha and introduce the filling before the varnish dries, for this reason: if the filling rests upon a thin foundation of gutta-percha, there will be sensation when the plug is submitted to hard biting. When the amalgam is packed before the varnish is dry, all is forced out except that which penetrates the dentine, or is used to fill up the roughened surface of the cavity. At all events, the filling rests firmly upon the dentine, and no circulation can go back of the filling. The amount remaining is so thin that shrinkage by loss of the solvent will amount to nothing.

In large cavities with thin walls in the molars, where the pulp is not in danger, I prefer oxychloride or oxyphosphate linings to gutta-percha, as they make a firm foundation. Oxyphosphate works well used as described for gutta-percha.

In this connection, I will say that a large phosphate filling, if inserted close upon the pulp, will in time destroy it. It may be years before this will occur; generally, the patient is warned in time to change the filling for gutta-percha, which will correct the difficulty; but, if allowed to continue after the filling has been worn at the edges or has commenced to decompose, the acids set free will devitalize the pulp. In no case have I seen any such action from cavity linings; there is little or no acid to be set free. The lamina is so very thin that it cannot be decomposed and leave a space. Decay rarely occurs around fillings thus lined. On removal of fillings thus inserted, it is almost impossible to separate the filling from the cavity-walls.

This lining is most essential in the use of the higher grades of amalgam, for the reason that amalgam that does not oxidize is no better than gold; it has no antiseptic properties like those contained in copper.

To explain, I will say that in the setting of telegraph-poles some companies wisely bore a hole in the pole above the ground and fill it with sulphate of copper; when the salt dissolves and is absorbed into the wood, more is added until the wood becomes saturated with the oxidized copper: wood in this condition does not decay.

This is precisely what occurs when porous dentine is filled with amalgam: the oxides and sulphides of the metals are absorbed into the surrounding dentine, taking the place of the lime salts and thus arresting decay.

Teeth in a normal condition are not discolored by amalgam fillings, because the structure is so dense that the oxides do not penetrate. The worst feature of amalgam is its shrinkage, which allows circulation and secondary decay. This is not offered as a new practice. It goes to show the importance of understanding the condition of the teeth in relation to the materials used.

I will close with this illustration, which is applicable to the subject before us: On entering a central telephone-office, one sees an immense switch-board with hundreds of brass-lined cavities, all numbered. It is the business of the operator to insert plugs into these cavities as ordered, on call. So long as the order is obeyed, all goes right. A mistake in plugging a cavity sends the current in the wrong direction. Such mistakes are often the cause of much irritation and not infrequently attended with vociferous reflex action.

The dentist, while on duty, has a switch-board before him; he should understand all combinations and be able to make the conditions, so as to secure the right results. Scientific knowledge of this nature is an element of success in saving teeth.

To recapitulate : Social, professional, physiological, and mechanical elements, combined, insure success.

Dentists are classed with the patients and practice that give them support.

The way to success is open and upward, with ample room above.

Progress is the outcome of associated efforts in the higher grades of practice.

Never limit the possibilities of others to the inability of self.

Stand firmly upon the foundation of true merit, without building upon the demerits of others.

Reading presents to the mind many theories; discussions help to obtain facts ; clinical instructions are seldom forgotten. Society membership is indispensable, socially, professionally, and protectively. In a literary way, read and take notes, reflect and record facts. In operating, regard each tooth as a patient; diagnose physiologically, prescribe with chemical adaptability, manipulate with mechanical accuracy, and there will be "no such word as fail."

